# *Besnoitia besnoiti* infection alters both endogenous cholesterol *de novo* synthesis and exogenous LDL uptake in host endothelial cells

**DOI:** 10.1038/s41598-019-43153-2

**Published:** 2019-04-30

**Authors:** Liliana M. R. Silva, Dieter Lütjohann, Penny Hamid, Zahady D. Velasquez, Katharina Kerner, Camilo Larrazabal, Klaus Failing, Carlos Hermosilla, Anja Taubert

**Affiliations:** 1 Institute of Parasitology, Biomedical Research Center Seltersberg, Justus Liebig University Giessen, Schubertstr. 81, D-35392 Giessen, Germany; 2 Institute for Clinical Chemistry and Clinical Pharmacology, University Clinics Bonn, Laboratory for Special Lipid Diagnostics/Center Internal Medicine/Building 26/UG 68, Sigmund-Freud-Str. 25, D-53127 Bonn, Germany; 3 Department of Parasitology, Faculty of Veterinary Medicine, Universitas Gadjah Mada, Jl. Fauna No. 2 Karangmalang, 55281 Yogyakarta, Indonesia; 4 Institute for Hygiene and Infectious Diseases of Animals, Justus-Liebig-University, Giessen, Frankfurter Str. 85-89, D-35392 Germany; 5 Unit for Biomathematics and Data Processing, Faculty of Veterinary Medicine, Justus Liebig University Giessen, Frankfurter Str. 95, D-35392 Giessen, Germany

**Keywords:** Parasite development, Parasite host response, Lipoproteins, Sterols

## Abstract

*Besnoitia besnoiti*, an apicomplexan parasite of cattle being considered as emergent in Europe, replicates fast in host endothelial cells during acute infection and is in considerable need for energy, lipids and other building blocks for offspring formation. Apicomplexa are generally considered as defective in cholesterol synthesis and have to scavenge cholesterol from their host cells for successful replication. Therefore, we here analysed the influence of *B. besnoiti* on host cellular endogenous cholesterol synthesis and on sterol uptake from exogenous sources. GC-MS-based profiling of cholesterol-related sterols revealed enhanced cholesterol synthesis rates in *B. besnoiti*-infected cells. Accordingly, lovastatin and zaragozic acid treatments diminished tachyzoite production.  Moreover, increased lipid droplet contents and enhanced cholesterol esterification was detected and inhibition of the latter significantly blocked parasite proliferation. Furthermore, artificial increase of host cellular lipid droplet disposability boosted parasite proliferation. Interestingly, lectin-like oxidized low density lipoprotein receptor 1 expression was upregulated in infected endothelial hostcells, whilst low density lipoproteins (LDL) receptor was not affected by parasite infection. However, exogenous supplementations with non-modified and acetylated LDL both boosted *B. besnoiti* proliferation. Overall, current data show that *B. besnoiti* simultaneously exploits both, endogenous cholesterol biosynthesis and cholesterol uptake from exogenous sources, during asexual replication.

## Introduction

*Besnoitia besnoiti* is an obligate intracellular apicomplexan parasite which causes bovine besnoitiosis and has a significant economic impact on cattle industry in endemic areas^1^. Clinical bovine besnoitiosis includes rather general signs during the acute febrile phase of infection (e. g. lethargy, tachycardia, tachypnoea, congestive mucosae, oedema, anorexia, weight loss) whilst massive skin alterations or bull infertility are characteristic for the chronic phase^2^. Successive reports on *B. besnoiti* infections in several European countries in the recent years^3–11^ revealed this disease as emerging in Europe^2,12^. During the febrile acute stage of besnoitiosis, tachyzoites mainly proliferate in bovine host endothelial cells of different organs and vessels causing vasculitis, thrombosis, and necrosis of venules and arterioles^2^. *In vitro* experiments proved a series of cell types besides endothelial cells as permissive for parasite replication and *B. besnoiti* showed fast proliferative qualities, which are alike to those of *Toxoplasma gondii* or *Neospora caninum*^4,13–16^. During acute proliferation, the parasite is in a significant need for energy and cell building blocks, which may either be scavenged from the host cell or be synthesized by the parasite itself, depending on its synthetic capacities. Especially for offspring production, the parasite needs vast amounts of cholesterol. Cholesterol was shown to be sequestered in cholesterol-rich organelles and to be inserted into the parasite plasma membrane and the parasitophorous vacuole membrane in the case of the closely related parasite *T. gondii*^17^. Furthermore, cholesterol is esterified for storage in lipid droplets, which were consistently found enhanced in apicomplexan parasites-infected host cells^17–28^. However, apicomplexan parasites are generally considered as defective in cholesterol synthesis^17,29–33^. For compensation, they need to scavenge cholesterol from their host cells thereby following different strategies of cholesterol acquisition. In general, two main routes of cholesterol disposal are provided by potential host cells: endogenous cholesterol *de novo* synthesis and sterol uptake from extracellular sources via specific receptors. These scavenging pathways are differentially exploited by different apicomplexan species. While several species, such as *T. gondii* (in Chinese hamster ovary cells - CHO), *Cryptosporidium parvum* or *N. caninum* mainly rely on host cellular LDL-mediated sterol uptake^17,33,34^, others mainly utilize host cellular *de novo* synthesis for cholesterol acquisition (e. g. *T. gondii* in macrophages)^35^. In contrast, hepatic *Plasmodium* spp. salvage cholesterol from both pathways but do not strictly depends on cholesterol acquisition for optimal proliferation^32^. Interestingly, the actual need of cholesterol of different apicomplexan species obviously depends on their mode of proliferation. Thus, for the slow but massively proliferating parasite *Eimeria bovis*, the simultaneous induction of both pathways was described^27,36,37^ whilst fast proliferating apicomplexan rather seem to utilize one single route of cholesterol acquisition. The fact that *T. gondii* triggers LDL-mediated sterol uptake in CHO cells but not in macrophages, where endogenous *de novo* synthesis represents the main source of cholesterol^17,35^, additionally strengthens the assumption that the mode of cholesterol acquisition may also depend on the host cell type.

To date, no data exist on the mode of cholesterol salvage being utilized by *B. besnoiti*. Therefore, the aim of the study was to analyse whether *B. besnoiti* infection of primary bovine endothelial host cells, i. e. the cell type that is mainly infected in the *in vivo* situation, influences the host cellular cholesterol *de novo* synthesis and exogenous sterol uptake, cholesterol conversion and esterification, as well as neutral lipid and lipid droplet formation during active intracellular proliferation. To provide actual data on the true cellular situation, we here analysed the content of several cholesterol-related sterols in *B. besnoiti*-infected endothelial host cells via a biochemical approach. Overall, the data show that *B. besnoiti* infections induce endogenous cholesterol synthesis rates in primary endothelial host cells and additionally profits from enhanced exogenous LDL levels for optimal parasite proliferation.

## Results

### *B. besnoiti* infections enhance total cholesterol contents in endothelial host cells

**Figure 1:**
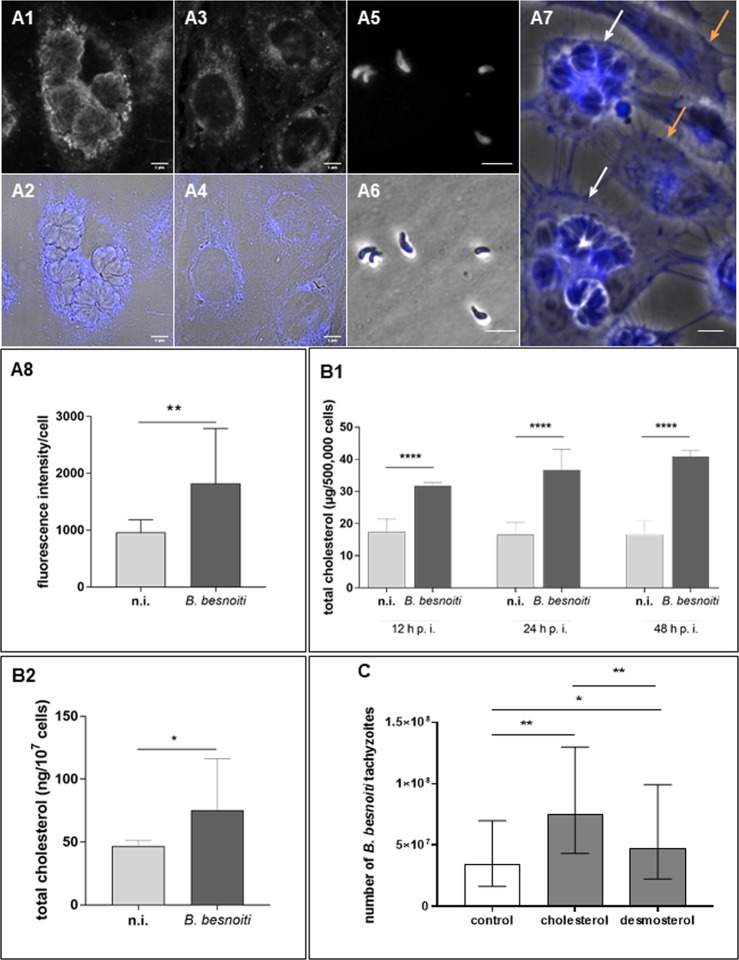
Cholesterol content in *B. besnoiti*-infected endothelial host cells and effects of cholesterol/desmosterol supplementation on parasite proliferation: (**A**) For cholesterol visualization, *B. besnoiti*-infected BUVEC and tachyzoite stages (24 h p. i.; A1-2, infected cell; A3-4, non-infected BUVEC; A5-6, *B. besnoiti* tachyzoites) were stained with filipin III (A1, A3 and A5); filipin + phase contrast (A2, A4, A6, A7). Single cell fluorescence intensity measurements were performed (A7; infected cells - white arrows; non-infected cells - orange arrows), and significantly increased amounts of cholesterol were observed in *B. besnoiti* infected cells (A8). (**B**) For analysis of total cholesterol content in *B. besnoiti*-infected host cells, BUVEC (*n* = 6) were infected with *B. besnoiti* tachyzoites and subjected to total cholesterol extraction using the Amplex Red test kit at different time points of infection (B1) or determined by GC-MS-based analyses (B2). Non-infected BUVEC were equally processed and served as negative controls. (**C**) To analyse the effect of exogenous cholesterol and desmosterol supplementation on tachyzoite production, *B. besnoiti*-infected BUVEC were either cultivated in non-supplemented (control) or cholesterol/desmosterol-enriched medium. 48 h after infection, the number of tachyzoites present in cell culture supernatants was determined. Geometric means of three biological replicates, geometric standard deviation, *p < 0.05, **p < 0.01, ***p < 0.001. Error bar 20 µm.

*B. besnoiti*-infected BUVEC (bovine umbilical vein endothelial cells) showed a stronger filipin staining (Fig. 1A1,A2) than non-infected controls (Fig. 1A3,A4) thereby suggesting a higher cholesterol content. Freshly released tachyzoites (Fig. 1A5,A6) were stained by filipin. Within tachyzoites, the strongest reactions were found in the posterior region of these stages. Single cell measurements of fluorescence intensity of filipin in *B. besnoiti*-infected (white arrows) and non-infected (orange arrows) host cells (Fig. 1A7, zoom of Supplementary Fig. 1) confirmed significantly enhanced levels of cholesterol in infected cells (*p* = 0.0024, Fig. 1A8). Amplex Red-based measurements of the total cholesterol contents (Fig. 1B1) confirmed a significantly increase of total cholesterol in *B. besnoiti*-infected endothelial host cells (effect of time: *p* = 0.033, infection: *p* = 0.0002 and interaction: *p* = 0.0014), as well as by GC-SM analyses (*p* = 0.0429; Fig. 1B2). Overall, kinetic analyses indicated increasing effects on cholesterol content with ongoing duration of infection leading to an enhancement of 1.8-fold, 2.2-fold and 2.4-fold at 12, 24 and 48 h p. i. (*t*-test with Bonferroni-Holm adjustment, *p* < 0.0001), respectively.

### Exogenous cholesterol and desmosterol supplementation boosts parasite proliferation

To control the role of exogenous cholesterol sources for parasite proliferation, we supplemented cholesterol and its precursor desmosterol by direct addition of the cell culture medium. As previously shown^38^, ethanol-dissolved cholesterol or desmosterol indeed directly acts on cells despite the hydrophobic characteristics of these molecules. In fact, supplementation with both desmosterol and cholesterol significantly enhanced *B. besnoiti* tachyzoite production in infected host cells (cholesterol *p* < 0.01; desmosterol *p* < 0.05; Fig. 1C). When comparing desmosterol and cholesterol for their effects, supplementation with cholesterol boosted parasite proliferation stronger than with desmosterol (*p* < 0.01). In addition, we supplied these molecules via MβCD complexes, in order to generate inclusion complexes that are able to donate these molecules to the host cell^39^. However, treating BUVEC with such complexes did not improve parasite proliferation (data not shown). In addition, treatments of tachyzoites with cholesterol-MβCD-complexes prior to infection also failed to influence parasite proliferation (data not shown).

### Neutral lipids and lipid droplet formation are enhanced in *B. besnoiti*-infected host cells

**Figure 2:**
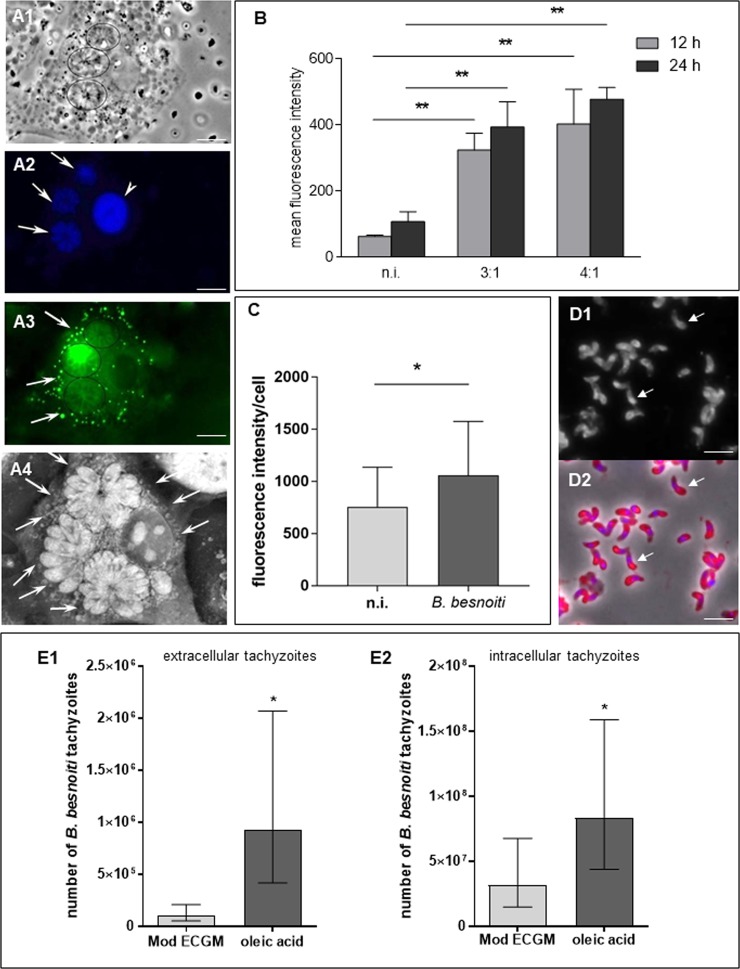
Neutral lipids and lipid droplet contents in *B. besnoiti*-infected endothelial host cells and effects of oleic acid treatments on parasite proliferation. (**A**) For lipid droplet visualization, *B. besnoiti*-infected endothelial host cells were stained by Bodipy 493/503 (A3) or directly analysed by tomographic microscopy (A4). Host cell nuclei were stained by DAPI (A2, arrowhead). A1-3: illustration of a single infected cell showing three typical *B. besnoiti* rosettes (24 h p. i., arrows) and a high abundance of cytoplasmic lipid droplets (A3, arrows). A4: 3D tomographic image of a *B. besnoiti* infected cell showing several cytoplasmic lipid droplets (arrows). (**B**) For lipid droplet quantification, *B. besnoiti*-infected BUVEC (MOI 3:1 and 4:1) were stained with Bodipy 493/503 at 12 (grey columns) and 24 h (black columns) p. i. and processed for flow cytometric analyses. Non-infected BUVEC were equally processed and served as negative controls. Arithmetic means of three BUVEC isolates, standard deviation (**p < 0.01). (**C**) For neutral lipid quantification, Nile Red-stained *B. besnoiti*-infected BUVEC (MOI: 3:1, 24 h p. i.) were analysed for fluorescence intensities on single cell level [single infected and non-infected single cells were estimated within the same cell layers under identical experimental conditions using the ImageJ software]. Data were calculated as arithmetic means ± standard deviation (*p = 0.0181). (**D**) Tachyzoite stages also showed strong reactions after Nile Red staining indicating the presence of neutral lipids. The strongest reactions were apparent in the posterior part of the tachyzoites (D1-2, arrows). (**E**) Effect of artificially enhanced lipid droplet disposability on *B. besnoiti* proliferation: to enhance lipid droplet formation in BUVEC, cells were treated with oleic acid in BSA-MβCD formulation prior to *B. besnoiti* tachyzoite infection. Non-treated BUVEC served as negative controls. Two days p. i. the number of tachyzoites being present in cell culture supernatants (E1) or still intracellular (E2) was estimated via PCR. Geometric means of three biological replicates, geometric standard deviation (*p < 0.05). Error bar 20 µm.

Neutral lipids and lipid droplets could be visualized via Bodipy 493/503 and Nile Red staining in *B. besnoiti*-infected BUVEC as well as in free tachyzoite stages. As estimated by fluorescence microscopy, infected cells showed increased numbers of lipid droplets in the cytoplasm of the host cell (Fig. 2A1-4). These findings were verified on a quantitative level via a FACS-based approach. By applying two different MOIs (3:1 and 4:1) we could show that lipid droplets are significantly enhanced in *B. besnoiti*-infected endothelial host cells when compared to non-infected controls (all *p* < 0.01, Fig. 2B). However, comparing the different MOIs or time points of infection, no significant differences were detected.

Microscopic analyses showed a stronger Nile Red staining in *B. besnoiti*-infected host cells (Supplementary Fig. 2; white arrows) when compared to non-infected cells within the same cell layer (Supplementary Fig. 2; orange arrows). Single cell measurements (from 3 different BUVEC isolates) confirmed significantly enhanced levels of neutral lipids in *B. besnoiti*-infected host cells (*p* = 0.0181, Fig. 2C). Tachyzoite stages also showed strong reactions after Nile Red staining indicating the presence of neutral lipids. As also shown for filipin staining, the strongest reactions were apparent in the posterior part of the tachyzoites (Fig. 2D1–2, arrows).

### Artificially enhanced lipid droplet disposability improves parasite proliferation

To estimate whether an increase of lipid droplet formation is beneficial for optimal parasite proliferation, we here artificially enhanced host cellular lipid droplet numbers by oleic acid treatments prior to infection. As depicted in Fig. 2E, the artificial enhancement of lipid droplet disposability indeed significantly boosted *B. besnoiti* tachyzoite production. Thus both, the number of freshly released (=extracellular, Fig. 2E1, *p* = 0.0109) and still intracellular (Fig. 2E2, *p* = 0.0259) tachyzoites (both log-transformed in the comparison) was found upregulated in oleic acid-treated BUVEC resulting in a 9-fold and 2.5-fold increase of parasite proliferation within 48 h, respectively.

### Live cell 3D holotomographic microscopy

**Figure 3:**
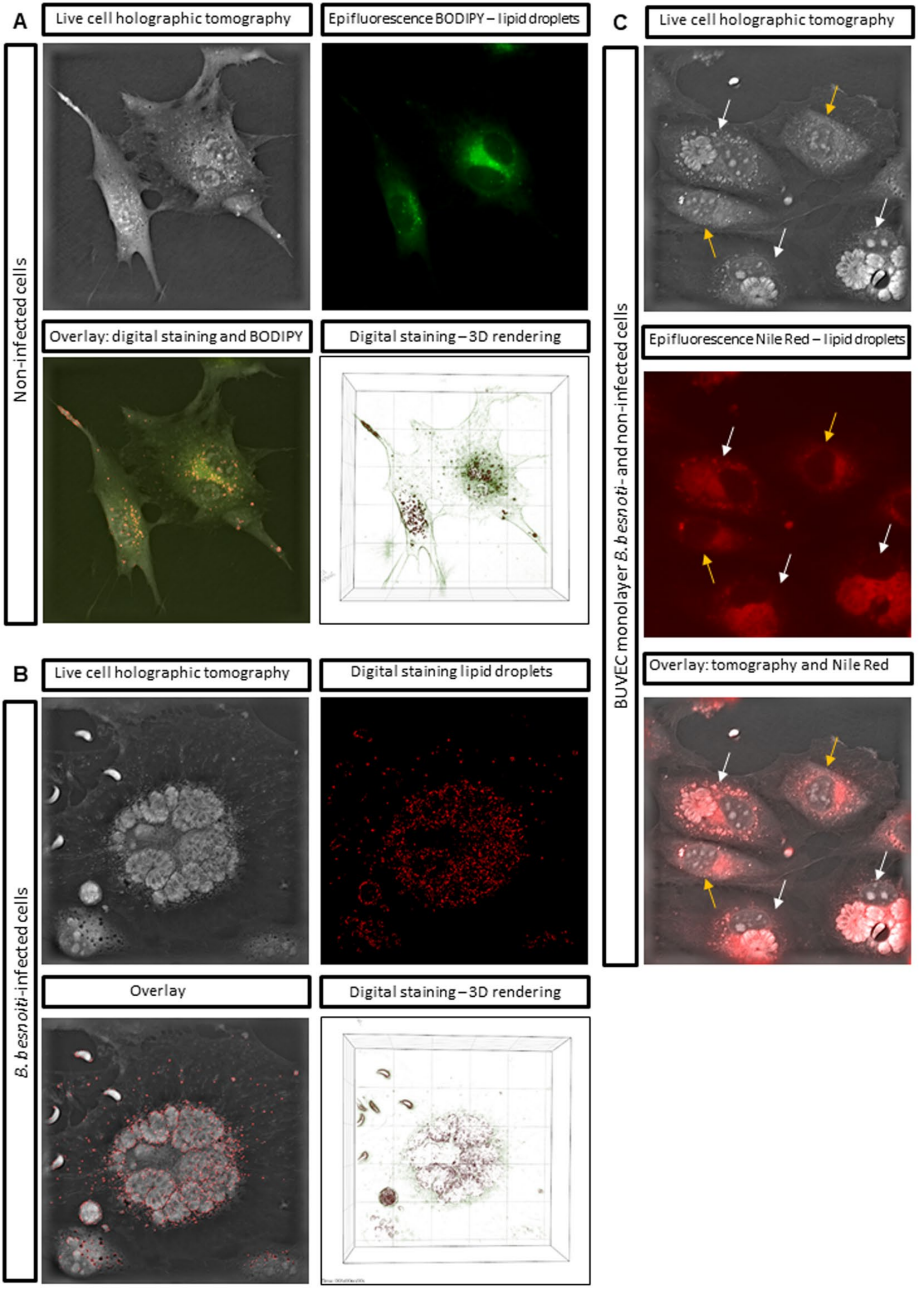
Live cell holographic tomography-based illustration of lipid droplets in non-infected and *B. besnoiti*-infected BUVEC. (**A**) Non-infected BUVEC were analysed for lipid droplet content via both, Bodipy 493/503-based staining (as visualized by epifluorescence) and live cell holographic tomography. 3D holotomographic images were obtained by using 3D cell-explorer microscope (Nanolive 3D Explorer) at 60X magnification (λ = 520 nm, sample exposure 0.2 mW/mm^2^) and a depth of field of 30 µm. Lipid droplets were stained via digital staining (STEVE software, Nanolive) according to the refractive index of the intracellular structures. Overlays from both detection techniques proved the applicability of tomographic microscopy via matching of the signals. **(B)** Holographic tomography of live *B. besnoiti*-infected BUVEC at 24 h p. i. and detection of lipid droplets via digital staining. **(C)** Holographic tomography of live *B. besnoiti*-infected (white arrows) and non-infected (orange arrows) BUVEC at 24 h p. i. and detection of lipid droplets via Nile Red staining.

3D holotomographic microscopy confirmed the presence of numerous lipid droplet-like structures in *B. besnoiti*-infected cells (Fig. 2A4, arrows). To prove the lipid droplet-nature of these structures, holographic tomography and epifluorescence analyses (Bodipy 493/503 staining) were performed in parallel on BUVEC which proved the precise matching of these two independent techniques (Fig. 3A). The mean refractive index (RI) of Bodipy stained lipid droplets was estimated (*n* = 50) and was 1.355 ± 0.00333. These parameters (RI > 1.355) were then applied to *B. besnoiti*-infected BUVEC (24 h p. i.) in 3D holotomographic microscopy and confirmed the presence of a high number of lipid droplets in infected cells (Fig. 3B). Moreover, Nile Red stained BUVEC also presented the same features (Fig. 3C).

### Cholesterol esterification is essential for optimal parasite proliferation

**Figure 4:**
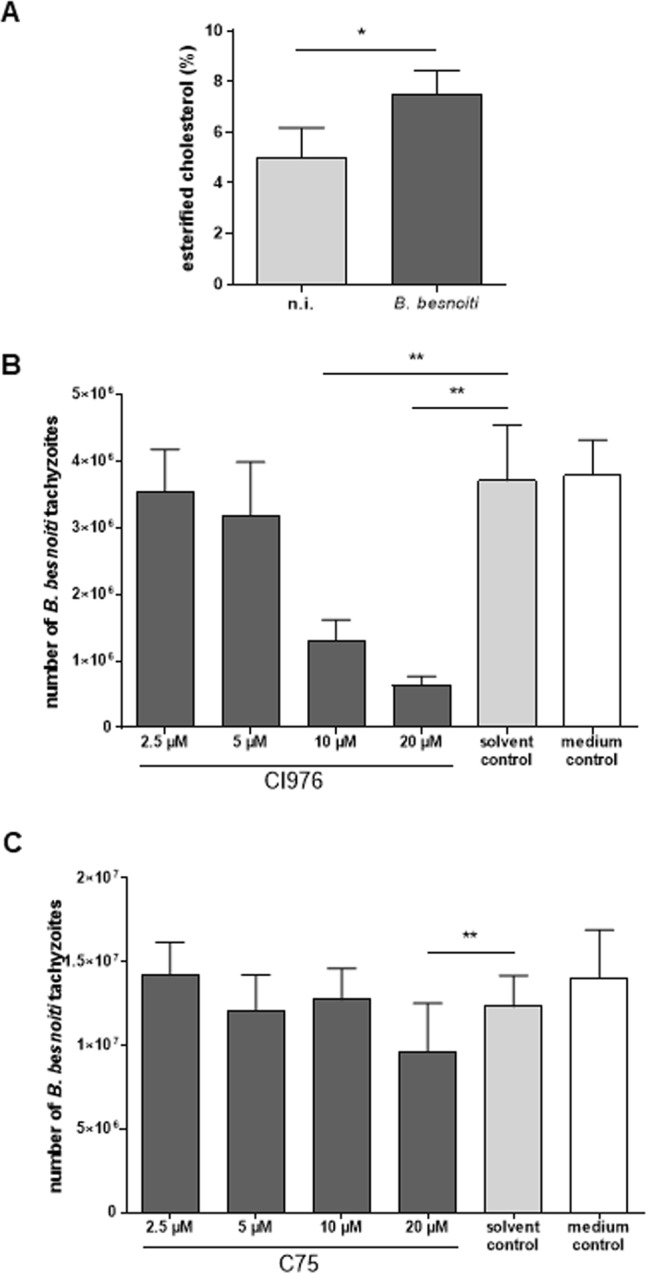
Cholesterol esterification in *B. besnoiti*-infected endothelial host cells and effects of CI976 and C75 treatments on parasite proliferation. (**A**) For estimation of the cholesterol esterification degree, *B. besnoiti*-infected BUVEC (*n* = 3) were subjected to GC-MS-based analyses of total and esterified cholesterol contents. Arithmetic mean of three biological with three technical replicates each, standard deviation; *p < 0.05. (**B**) Effects of CI976 (inhibitor of cholesterol esterification) treatment on tachyzoites proliferation: BUVEC were treated with CI976 (2.5, 5, 10 and 20 µM) 24 h before *B. besnoiti* infection. Non-treated host cells served as controls. 48 h after infection, the number of tachyzoites present in cell culture supernatants were measured. Bars represent arithmetic means of three biological replicates, standard deviation (**p < 0.01). (**C**) Effects of C75 (inhibitor of fatty acids synthesis) treatment on *B. besnoiti* replication. BUVEC were treated with C75 (2.5, 5, 10 and 20 µM) 24 h before *B. besnoiti* infection. Non-treated host cells served as controls. 48 h after infection, the number of tachyzoites present in cell culture supernatants was measured. Bars represent arithmetic means of three biological replicates, standard deviation (**p < 0.01).

Lipid droplets represent the main storage organelles for esterified cholesterol. Given that lipid droplets were found enhanced in *B. besnoiti*-infected BUVEC, we here analysed by biochemical means whether esterified cholesterol content was upregulated by *B. besnoiti* infection. Referring to total cholesterol content, *B. besnoiti*-infected BUVEC indeed showed a 1.5-fold, significant increase of esterified cholesterol levels when compared to non-infected controls (Fig. 4A, *p* = 0.045), thereby indicating infection-induced enhancement of free cholesterol conversion via esterification. To analyse further the role of cholesterol esterification for parasite proliferation we additionally performed functional inhibition experiments by the use of CI976, an inhibitor of the cholesterol-esterifying enzyme, sterol O-acyltransferase. Overall, CI976 treatments effectively inhibited *B. besnoiti* proliferation in a dose-dependent (*p* = 0.0038) manner (10 μM and 20 µM treatments: both *p* < 0.01; Fig. 4B). Thus, CI976 treatments led to a reduction of tachyzoite production of 4.6%, 14.1%, 64.7% and 82.9% when the cells were treated with 2.5, 5, 10 and 20 µM CI976 (Fig. 4B). Based on the inhibition of tachyzoite production, an IC_50_ of 7.56 µM was calculated for CI976 treatments. Microscopic control revealed that the host cells themselves were not altered in their morphology by CI976 treatments.

For cholesteryl ester formation, the hydroxyl group of cholesterol is linked to the carboxylate group of a fatty acid. Therefore, we additionally tested whether the blockage of fatty acid synthesis by the compound C75 would also impair *B. besnoiti* proliferation. C75 treatments of *B. besnoiti*-infected host cells moderately inhibited tachyzoite formation in dose dependent reduction (*p* = 0.049). However, only at 20 µM concentration a significant reduction of the number of parasites was observed when compared to solvent control (DMSO 0.1%; *p* = 0.0032; Fig. 4C).

### Endogenous cholesterol synthesis is enhanced in endothelial host cells and appears essential for optimal *B. besnoiti* proliferation

Endogenous *de novo* cholesterol synthesis is performed by a multi-step biochemical pathway being supported by numerous enzymatic reactions. Given that analyses on gene transcription or protein expression of certain involved molecules may not precisely reflect their true enzymatic activity, we here analysed the actual content of cholesterol-related sterols (e. g. cholesterol precursors, metabolites) via biochemical means in *B. besnoiti*-infected BUVEC and control cells. Therefore, the content of three groups of cholesterol-related sterols were measured: *i)* biosynthetic precursors of cholesterol in the endogenous synthesis pathway serving as indicators of cellular cholesterol *de novo* synthesis (lanosterol, dihydrolanosterol, lathosterol 7-dehydrocholesterol desmosterol); *ii)* downstream metabolites of *de novo* synthesis and indicators of cholesterol conversion [cholestanol, 24-hydroxycholesterol (24-OHC), 25-hydroxycholesterol (25-OHC), 27-hydroxycholesterol (27-OHC), 7α-hydroxycholesterol (7α-OHC), 7-ketocholesterol (7-ketoC), 4β-hydroxycholesterol (4β-OHC) and 7β-hydroxycholesterol (7β-OHC)] with some of these molecules (e. g. 7-ketoC, 7α-OHC and 7β-OHC) being recognized as indices of cellular oxidative stress; *iii)* phytosterols as indicators of cholesterol-uptake from the extracellular environment (campesterol, stigmasterol and sitosterol). We here calculated sterol:cholesterol ratios (Table 1) which are generally accepted as indicative for endogenous cholesterol synthesis rates. Notably, the individual BUVEC isolates differed considerably in their basic absolute levels of sterols as indicated by rather high standard deviations already present in non-infected samples.

**Table 1:** Ratios of sterol/oxysterol to cholesterol (GC-MS-based analyses) in *Besnoitia*-infected and non-infected BUVEC (*n* = 3).

Molecule	sterol:cholesterol ratio in control cells	sterol:cholesterol ratio in infected cells	n-fold	t-test *p*-value
**Lanosterol**	0.323 ± 0.133	0.863 ± 0.158	2.67	**<0.0001**
**Dihydrolanosterol**	0.035 ± 0.014	0.049 ± 0.009	1.41	**0.0071**
**Lathosterol**	7.718 ± 3.517	14.710 ± 3.867	1.91	**0.0007**
**7-Dehydrocholesterol**	3.263 ± 0.416	5.765 ± 1.661	1.77	**0.0008**
Desmosterol	3.383 ± 2.179	3.861 ± 1.069	1.14	n.s.
Cholestanol	2.680 ± 0.312	2.626 ± 0.363	0.98	n.s.
7α-OH Cholesterol	0.124 ± 0.055	0.129 ± 0.020	1.04	n.s.
24-OH Cholesterol	0.131 ± 0.065	0.104 ± 0.029	0.79	n.s.
25-OH Cholesterol	0.094 ± 0.105	0.054 ± 0.025	0.57	n.s.
**27-OH Cholesterol**	0.077 ± 0.041	0.048 ± 0.006	0.62	**0.0368**
4β-OH Cholesterol	0.086 ± 0.051	0.064 ± 0.013	0.75	n.s.
7β-OH Cholesterol	0.239 ± 0.089	0.257 ± 0.059	1.07	n.s.
7-keto Cholesterol	2.021 ± 1.352	1.655 ± 0.539	0.82	n.s.
Campesterol	0.195 ± 0.024	0.173 ± 0.046	0.89	n.s.
**Stigmasterol**	0.101 ± 0.034	0.075 ± 0.017	0.73	**0.0105**
**Sitosterol**	0.254 ± 0.058	0.189 ± 0.047	0.74	**0.0118**

n.s. not significant.

Overall, concerning indicators of endogenous cholesterol synthesis, *B. besnoiti* infections indeed led to a shift of sterol:cholesterol ratios by that way that several cholesterol precursors were found at enhanced contents in parasite-infected BUVEC. Overall these reactions proved significant for lanosterol (*p* < 0.0001), dihydrolanosterol (*p* = 0.0071), lathosterol (*p* = 0.0007) and 7-dehydrocholesterol (*p* = 0.0008) and indicated that host cellular *de novo* synthesis of cholesterol is upregulated by *B. besnoiti* infection (Table 1). In contrast to indicators of endogenous cholesterol synthesis, most oxysterols representing downstream metabolites were not found changed in their sterol:cholesterol ratios. Thus, oxysterol:cholesterol ratios denied any positive shift of these ratios in infected cells, but even confirmed a slight decrease of the 27-OHC:cholesterol ratio (*p* = 0.037) in infected cells (Table 1). Given that especially 7β-OHC, 7-ketoC and 7α-OHC upregulation may indicate oxidative cell stress reactions since these molecules represent autoxidatives that are mainly formed by radical oxidative species, *B. besnoiti* infections do not appear to cause considerable oxidative stress in bovine endothelial host cells. Along with enhanced endogenous cholesterol *de novo* synthesis, cholesterol-related needs can also be satisfied via an enhanced sterol uptake from the extracellular environment. Since phytosterols exclusively are of plant origin and are submitted to the cells via the FCS fraction, intracellular phytosterols levels are often used as indices of sterol uptake. Although the overall absolute levels of sitosterol, stigmasterol and campesterol were found slightly increased in *B. besnoiti*-infected host cells (sitosterol: 1.2-fold, stigmasterol: 1.4-fold and campesterol: 1.5-fold), phytosterol:cholesterol ratios did not confirm enhanced levels and even showed slightly reduced values for stigmasterol (*p* = 0.0105) and sitosterol (*p* = 0.0118) (Table 1) in *B. besnoitia*-infected cells.

**Figure 5:**
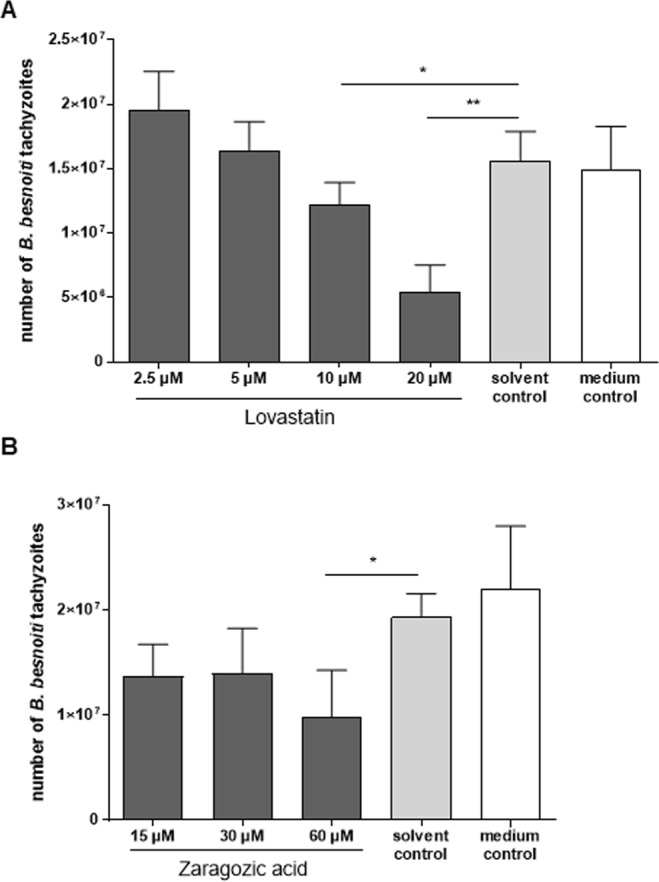
Effects of lovastatin (**A**) and zaragozic acid (**B**) treatments on *B. besnoiti* tachyzoite production. BUVEC were treated with lovastatin (**A**) or zaragozic acid **(B)** 24 h before *B. besnoiti* infection. Non-treated host cells served as controls. 48 h after infection, the number of tachyzoites present in cell culture supernatants was measured. Bars represent arithmetic means of three biological replicates, standard deviation (*p < 0.05; **p < 0.01).

Given that cholesterol-related sterol profiling indicated enhanced endogenous cholesterol synthesis rates, we here additionally performed functional inhibition experiments using blockers of the mevalonate biosynthesis pathway. Therefore, statin treatments were here applied. Statins represent a class of drugs widely used to lower plasma cholesterol levels^40^. We used lovastatin, which affects the total cellular isoprenoid/steroid synthesis and thus interferes at a very early step of *de novo* synthesis by blocking HMG-CoA-reductase (HMGCR). Overall, lovastatin treatments exhibited dose-dependent significant effects on parasite proliferation (*p* = 0.02). Thus, tachyzoite production was reduced for 21% and 66% when infected cells were treated with 10 µM (*p* < 0.05) and 20 µM (*p* < 0.01) lovastatin, respectively (Fig. 5A). Based on the inhibition of tachyzoite production an IC_50_ of 11.31 µM was calculated for lovastatin treatments.

Besides lovastatin, we also used zaragozic acid (syn. squalestatin) for treatments of *B. besnoiti*-infected BUVEC. Zaragozic acid is a squalene synthase inhibitor, which directly targets sterol synthesis. Zaragozic acid treatments resulted in a reduction of parasite replication (Fig. 5B), but effects were less prominent than those observed with lovastatin and required higher inhibitor concentrations. However, 60 µM zaragozic acid treatment induced a significant reduction of *B. besnoiti* replication (48.8%, *p* < 0.05). These data underline the key role of cellular *de novo* synthesis for successful parasite replication.

### *B. besnoiti* infections induce LOX-1 but not LDLR expression and profit from exogenous sterol uptake

**Figure 6:**
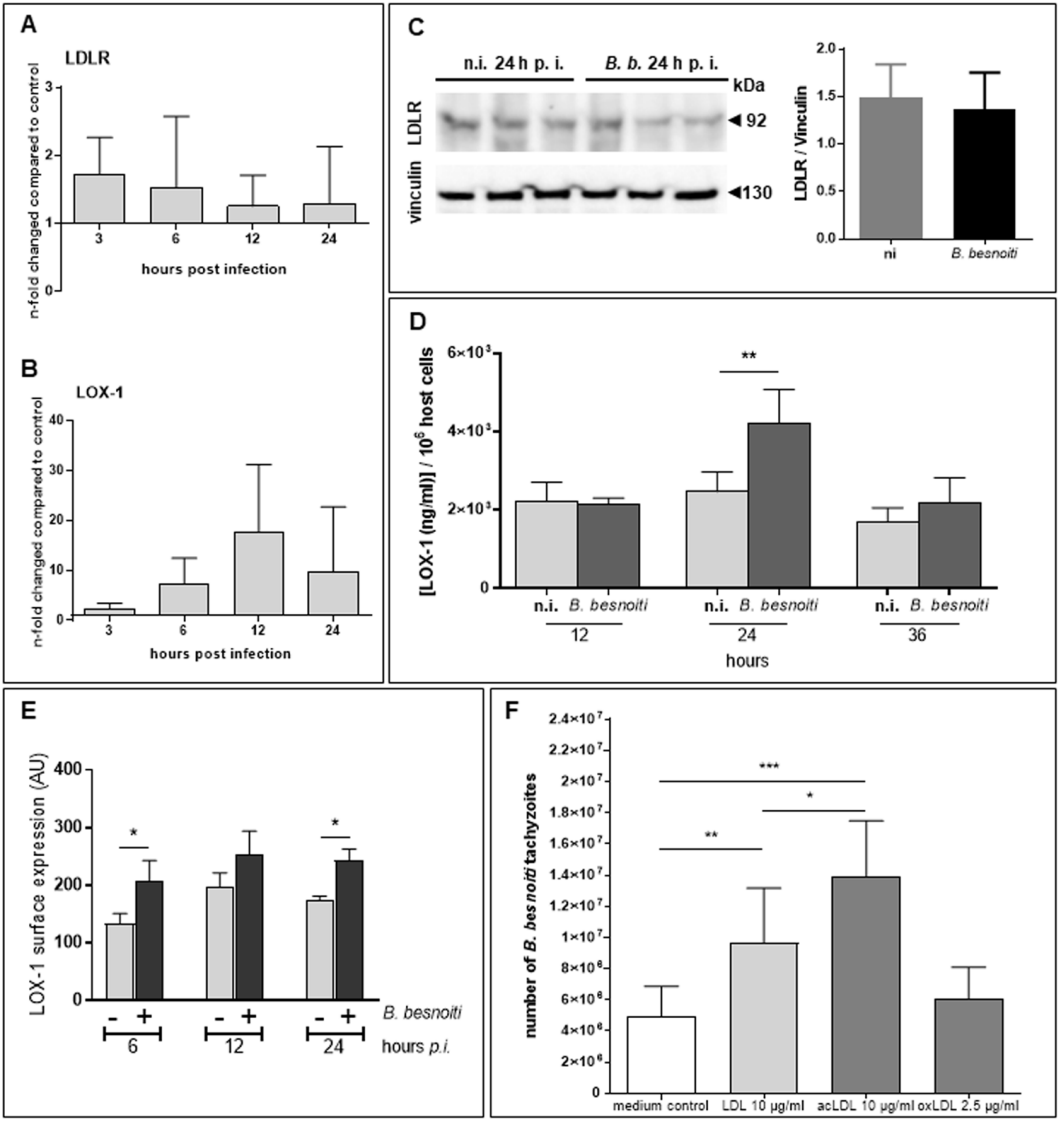
LDLR and LOX-1 gene transcription and protein expression in *B. besnoiti*-infected endothelial host cells and effects of LDL supplementation on parasite proliferation. (**A**,**B**) For estimation of LDLR and LOX-1 gene transcription during *B. besnoiti* replication *in vitro*, total RNA of infected and non-infected BUVEC (*n* = 3) was extracted at different time points p. i., reverse transcribed and submitted to LDLR- and LOX-1-specific real-time qPCR. Data represent arithmetic means ± standard deviation. (**C**) For analyses on protein expression of LDLR, *B. besnoiti*-infected and non-infected BUVEC soluble protein fractions were isolated from cell pellets at 24 h p. i. and submitted to immunoblotting using LDLR-specific antibodies. The expression of vinculin in each sample was used for protein content normalization. Two different gels/blots from the same samples. (**D**,**E**) For analyses on protein expression of LOX-1, *B. besnoiti*-infected BUVEC and non-infected controls were either analysed by a commercial test kit (LOX-1 bovine ELISA kit, DL Develop) (**D**) or subjected to flow cytometric analyses on LOX-1-related surface expression (**E**) by using LOX-specific antibodies. Bars represent arithmetic mean of three biological replicates ± standard deviation (*p < 0.05; **p < 0.01). (**F**) For LDL supplementation experiments, non-modified LDL (LDL), acetylated LDL (acLDL) or oxidised LDL (oxLDL) were supplemented at indicated concentrations to *B. besnoiti*-infected and non-infected host cell cultures. The total number of *B. besnoiti* tachyzoites was determined at 48 h p. i. Arithmetic mean ± standard deviation of three biological replicates (*p < 0.05, **p < 0.01, ***p < 0.001).

Besides being synthesized endogenously, cholesterol may also be taken-up from extracellular sources via receptor-mediated LDL incorporation. The endothelial cell type is well-known to internalize different LDL species, such as non-modified LDL (LDL), acetylated LDL (acLDL) or oxidized LDL (oxLDL). LDL uptake is preferentially mediated by the classical LDL receptor (LDLR) whilst a series of non-classical, so-called scavenger receptors (e. g. LOX-1, SRB1) preferentially promote modified LDL incorporation. In this study we focused on two key receptors, LDLR and LOX-1, and analysed whether the gene transcription and protein expression of these receptor was influenced by *B. besnoiti* infections in BUVEC. Gene transcriptional profiling revealed that LDLR and LOX-1 were differentially altered by parasite infection. Whilst LDLR gene transcription and protein expression was not altered in *B. besnoiti*-infected BUVEC (Fig. 6A,C), LOX-1 gene transcripts were found upregulated throughout *B. besnoiti in vitro* infection peaking at 12 h p. i. (Fig. 6B). In agreement, LOX-1 protein expression was also found upregulated in infected cells: this was confirmed by two methods, a commercial LOX-1-specific ELISA (Fig. 5D, *B. besnoiti* infection vs. controls at 24 h p. i.: *p* = 0.0025) and by the FACS-based measurement of LOX-1 surface expression (Fig. 5E; *B. besnoiti* infection vs. controls; 6 h p. i.: *p* = 0.0167, 24 h p. i.: *p* = 0.0243).

Given that LDLR and LOX-1differentially promotes LDL species uptake, we here analysed whether supplementation of different LDL species (LDL, acLDL, oxLDL) is beneficial for parasite proliferation. Thus, LDL and acLDL was supplemented to *B. besnoiti*-infected BUVEC cultures at 10 µM final concentration. In case of oxLDL, the final concentration had to be reduced to 2.5 µM since this LDL variant revealed as toxic for BUVEC at higher concentrations. As depicted in Fig. 5F, both LDL and acLDL significantly boosted *B. besnoiti* tachyzoite production when compared to non-supplemented controls (LDL: *p* < 0.01; acLDL: *p* < 0.001). Moreover, acLDL induced a significantly stronger parasite proliferation than LDL supplementation (*p* < 0.05). In contrast, oxLDL failed to improve parasite replication at 2.5 µM supplementation. These data revealed that optimal *B. besnoiti* proliferation depends on the supply of exogenous cholesterol or other lipids and additionally proves that infected host cells may profit from different LDL variants, i. e. from modified and non-modified LDL.

## Discussion

Apicomplexan parasites are generally considered as defective in cholesterol synthesis. Given that apicomplexan species are obligate intracellular parasites with highly proliferative capacities, this metabolic characteristic renders these pathogens as highly dependent on their respective host cells in terms of cholesterol supply. In principle, host cells support two major pathways of cholesterol resourcing: endogenous *de novo* synthesis and exogenous sterol uptake. Most reports indicate that different apicomplexan species may utilize diverse pathways of cholesterol acquisition in a species- or even host cell type-dependent manner.

The current data demonstrate that *B. besnoiti* uses both pathways of cholesterol acquisition when infecting bovine endothelial cells, which correspond to host cells to be infected during the acute phase of cattle besnoitiosis^2,41^. This is in agreement to recent data on a slow proliferating apicomplexan species, *Eimeria bovis*^27,36,37^ but differs from findings on *T. gondii*^17^. Since we here measured the cellular content of cholesterol biosynthetic precursors and metabolites via GC-MS-based approaches, the current data should directly reflect the actual biochemical situation in *B. besnoiti*-infected endothelial host cells. Sterol:cholesterol ratios of cholesterol biosynthetic precursors, which are generally accepted as reflecting the activity of the endogenous cholesterol biosynthetic pathway^42^, showed a significant enhancement in *B. besnoiti*-infected host cells. Accordingly, an infection-driven increase of lanosterol-, dihydrolanosterol-, 7-dehydrocholesterol- and lathosterol-related ratios was detected. In line, a pivotal role of cholesterol *de novo* synthesis was also suggested for *E. bovis* or *T. gondii* infections^27,35,36,43^ and in case of *E. bovis* these data also relied on GC-MS-based analyses^37^. These biochemical data were furthermore supported by a significant reduction of *B. besnoiti* proliferation triggered by statin treatments, which interfere with endogenous cholesterol synthesis. Statins represent a class of drugs widely used to lower plasma cholesterol levels^40^. Statin treatments also proved effective in other apicomplexan-related infection systems, such as *T. gondii*-infected macrophages^35,43^, *Plasmodium*- and *Babesia*-infected erythrocytes^44^, *C. parvum*-infected epithelial cells^33^ or *E. bovis*-infected BUVEC^36^. The current data revealed a higher efficacy of lovastatin, which interferes with the total cellular isoprenoid/steroid synthesis, when compared to zaragozic acid that directly targets sterol synthesis. Noteworthy, the blockage of merozoite production in other apicomplexan also depends on the choice of statin. As such, treatments with rosuvastatin and atorvastatin failed to influence parasite proliferation, whilst the application of lovastatin in higher concentrations (~72–96 µM) reduced tachyzoite production for more than 50% in *T. gondii*-infected macrophages^43^. Zaragozic acid is an inhibitor of squalene synthase and for this reason considered as more specific for cholesterol blockage than other statin treatments^45^. In *B. besnoiti*-infected host cells, 60 µM zaragozic acid treatments resulted in a significant blockage of tachyzoites production (reduction of 48.8%) confirming that *B. besnoiti* replication depends on host cell *de novo* synthesis. In contrast to *E. bovis* with 70.2% reduction rate at 5 µM treatment^36^, 15 µM zaragozic acid treatments in *C. parvum*-infected epithelial cells only induced moderate effects, as indicated by 25% growth delay^33^. Comparable rates of reduction were described in *T. gondii*-infected macrophages with 1–10 µM zaragozic acid^35^. Squalene synthase-defective CHO cells revealed no significant anti-proliferative effects on *T. gondii* development compared to non-defective controls^17^. However, other authors applied two quinuclidine-based inhibitors of squalene synthase in *T. gondii*-infected epithelial cells and described anti-proliferative effects of both compounds achieving a similar percentage of reduction of tachyzoite replication as in *B. besnoiti*-infections (48–58% reduction) but with much lower doses of zaragozic acid (3 µM)^46^. Overall, the data on cholesterol biosynthetic precursors and inhibitor treatments indicate that successful *B. besnoiti* replication depends on the host cell cholesterol *de novo* synthesis. Nevertheless, the fact that replication is not entirely blocked by zaragozic acid treatment may argue for additional sources of cholesterol besides *de novo* synthesis.

As a general feature, sterol up-take from the extracellular compartment seems to be exploited by apicomplexan parasites in a species-specific and host cell-specific manner. Whilst this pathway appeared of major importance in the case of *T. gondii* in CHO cells^17^, *C. parvum* in epithelial cells^33^, *N. caninum* infections^34^ or *E. bovis*-infected endothelial cells ^37^, a minor or even absent role of LDL-mediated cholesterol supply was reported for *T. gondii* infections in macrophages^35^ or for *Plasmodium* spp. infections in hepatocytes^32^. In the case of *B. besnoiti* infections, the current data on LDL supplementation confirm a pivotal role of exogenous sterol uptake for parasite proliferation. For the first time, we here applied different LDL species (LDL, acLDL, oxLDL), which are present in blood or lymph and may therefore all serve as exogenous sources *in vivo*. Interestingly, the supplementation with both, acLDL and LDL, revealed as beneficial for *B. besnoiti* proliferation and boosted tachyzoite production. LDL supplementation was also beneficial for *T. gondii* proliferation in CHO cells^17^ whilst such a treatment had no stimulatory effects on hepatic *Plasmodium* spp. and *C. parvum* proliferation^32,33^ or on *T. gondii* growth in macrophages^35^ indicating parasite- and cell type-specific mechanisms. However, to our best knowledge this signifies the first report on acLDL-mediated effects on intracellular apicomplexan proliferation.

In addition to LDL-related data, the direct exogenous supply of excess cholesterol or desmosterol also boosted *B. besnoiti* proliferation. This is in line with other data that proved this method of cholesterol supplementation as effective in the case of *T. gondii* in CHO cells^17^ or endothelial *E. bovis* infections^27^. So far, it remains unclear why the supplementation of cholesterol via MβCD complexes failed to influence parasite proliferation which contrasts data of other studies^47^. Referring to exogenous sterol supplementation, the current data on phytosterols measurements appear somewhat conflicting. Phytosterols signify cholesterol analogues that exclusively originate from plant dietary intake^48^. In cell cultures, these molecules are derived from FCS present in the cell medium and an enhanced cellular content of plant sterols is commonly accepted as an indicator of cellular sterol uptake from the extracellular environment^42,48^. However, current biochemical measurements data failed to show enhanced phytosterol:cholesterol ratios in *B. besnoiti*-infected BUVEC but even revealed slightly reduced values for stigma- and sitosterol. So far, we have no plausible explanation for these findings. While cell type-specific differences in individual phytosterol species uptake were reported, the capacity of BUVEC to incorporate all three phytosterols here detected was previously proven since a significant enhancement of sitosterol, stigmasterol and campesterol contents was reported in *E. bovis*-infected endothelial cells ^37^.

Given that LDL species are internalized via a receptor-mediated uptake, we here furthermore analysed whether *B. besnoiti* infections influence the gene transcription and protein expression of two classical endothelial LDL-related receptors. In contrast to reports on *T. gondii* infections in CHO cells^17^ and *E. bovis* infections in BUVEC^27^, LDLR expression was not altered by *B. besnoiti* infections. These data were in accordance to the fact that reduced LDLR expression did not affect the liver stage burden in the case of *Plasmodium* spp.^32^. Contrary to LDLR, an infection-driven upregulation of the scavenger receptor LOX-1 was here found in *B. besnoitia*-infected BUVEC on both, gene transcriptional and protein expression level. Recent microarray data on *B. besnoiti*-infected BUVEC confirmed a significant upregulation of LOX-1 (Silva L.M.R., unpublished data). LOX-1 is considered as an important receptor for ox-LDL internalization in vascular endothelial cells^49–51^ but is also able to bind acLDL at a comparable affinity^52^. This receptor was recently also reported to be upregulated in *E. bovis* infections of bovine endothelial cells^27,53^. Given that in both cases of LOX-1 induction, endothelial host cells served as host cells may indicate a cell type-specific mechanism.

Excess cellular levels of free cholesterol demand for cholesterol efflux or conversion since too high concentrations are toxic for cells. Two main conversion routes in endothelial cells involve cholesterol esterification and oxidation. In the current study, a significantly enhanced level of esterified cholesterol was detected in *B. besnoiti*-infected cells when compared with non-infected controls. This corresponds to recent findings in *E. bovis*-infected BUVEC^37^. In line, the key role of cholesterol esterification was here additionally confirmed by its chemical blockage via CI976 treatments leading to a significant inhibition of *B. besnoiti* replication. Thus, tachyzoite production was reduced by 64.7 and 82.9%, at 10- and 20-µM treatments, respectively. These data are in agreement with reports on *T. gondii* and *E. bovis* documenting the essential role of cholesterol esterification for optimal parasite proliferation^19,28^. Concerning relevant inhibitor concentrations, it has to be noted that antiproliferative effects occurred at a comparable level in *T. gondii* (merozoite reduction rates of approximately 60 and 70% induced by 4- and 10-μM CI976 treatments) and *B. besnoiti* infections. Even stronger effects were reported for *E. bovis* infections since 5 µM treatments almost entirely inhibited merozoite I production (99.6% reduction) as mirrored by a low IC_50_ of 0.34 µM^36^. The higher sensitivity of *E. bovis* to CI976 treatments may be due to a stronger need for cholesteryl ester formation during macromeront formation (>120.000 merozoites I) when compared to non-macromeront-forming parasites. Interestingly, *T. gondii* appears to be able to synthesize and store cholesteryl esters by itself, if host cell cholesterol is available^19,28^. In fact, two SOAT-like molecules were identified in *T. gondii* stages and proved sensitive to SOAT inhibitor treatments^28,54^. So far, no data are available with this respect for *B. besnoiti*. Nevertheless, it remains to be elucidated whether CI976-driven, detrimental effects on *B. besnoiti* proliferation accounted only to the host cell compartment or were also brought about by direct antiparasitic effects, as in the case of *T. gondii*.

Cholesteryl esters play a pivotal role in the development of several apicomplexan parasites and cholesterol and fatty acids are needed for cholesteryl ester formation, though the effects of fatty acid synthase blockage was additionally here investigated. The synthetic α-methylene-γ-butyrolactone compound C75 inhibits fatty acid synthase activity and has been studied for its anti-inflammatory and anti-tumoral activities^55–57^. C75 treatments induced a significant reduction of *B. besnoiti* replication at 20 µM concentration. However, in the case of *E. bovis*, lower concentrations of C75 were needed for anti-proliferative effects indicating a high relevance of fatty acids for this parasite^36^. Moreover, C75 inhibition was ineffective on *Trypanosoma cruzi* growth in infected macrophages^58^.

The main storage organelles of cholesteryl esters are represented by lipid droplets. The key role of enhanced lipid droplet formation in infected host cells was reported for several protozoan parasites, such as *T. cruzi*, *T. gondii*, *P. berghei*, *P. falciparum* or *E. bovis*^17–23,25–28^ and was here confirmed for *B. besnoiti* infections. Thus, a significantly enhanced abundance of lipid droplets was found in *B. besnoiti*-infected BUVEC. The pivotal role of these organelles was additionally confirmed by the fact that an artificial increase of lipid droplet disposability via oleic acid treatments significantly boosted tachyzoite formation. Likewise, an oleic acid-driven boost of offspring production was also observed in case of *E. bovis*, corroborating the assumption of lipid droplets mainly functioning as lipid storage and “feeder” organelles in *E. bovis* macromeront formation^27^.

Overall, *B. besnoiti* infections failed to trigger the synthesis of oxysterols in host endothelial cells. Interestingly, enzymatically synthesized side-chain oxysterols (e. g. 24-OHC, 25-OHC, 27-OHC), which are known as key regulators of cholesterol homoeostasis and as effector molecules of cellular innate immunity^59–63^ were not affected by *B. besnoiti* infections. In contrast, *E. bovis* infections were recently proven to selectively upregulate the synthesis of 25-OHC, a molecule that was proven to bear antiparasitic properties^37^. Moreover, a lack of 7β-OHC, 7-ketoC and 7α-OHC upregulation, which is generally mediated via autoxidative processes, revealed that BUVEC do not experience considerable oxidative cell stress triggered by *B. besnoiti* infections.

In summary, this investigation adds further data on the modulatory capacity of the fast proliferating apicomplexan parasite *B. besnoiti*. The current data strengthen the assumption that the modulation of distinct pathways of cholesterol acquisition is dependent on both, the parasite species and the host cell type. Thus, we here show that successful *B. besnoiti* infections in primary bovine endothelial host cells  rely on both, endogenous cholesterol synthesis and on sterol uptake from exogenous sources by using different LDL species leading to selective LOX-1 upregulation.

## Materials and Methods

### Host cell culture

Primary bovine umbilical vein endothelial cells (BUVEC) were isolated from bovine umbilical cords as recently described^64^. For cell cultures, BUVEC were resuspended in complete ECGM (PromoCell) in 75 cm^2^ plastic tissue culture flasks (Greiner BioOne). After confluency, cells were split, plated in 25 cm^2^ plastic tissue culture flasks (Greiner BioOne) and incubated at 37 °C and 5% CO_2_ atmosphere. Medium was changed every 2–3 days using ECGM-medium supplemented with 70% medium 199 (ModECGM); 500 U/mL penicillin (Sigma-Aldrich) and 50 μg/mL streptomycin (Sigma-Aldrich) and 10% FCS (Biochrom)^65^. Only cells of low passages (1–3) were used for this study.

### Parasites

*Besnoitia besnoiti* (strain Bb1Evora04) tachyzoites were maintained by serial passages in *Mycoplasma*-free BUVEC according to previous reports^41^. Confluent BUVEC monolayers in 25 cm^2^ flasks were infected with freshly isolated *B. besnoiti* tachyzoites (MOI = 5:1). Free-released tachyzoites were collected from BUVEC culture supernatants, washed in ModECGM and pelleted (400 × *g*, 12 min). *B. besnoiti* tachyzoites were counted in a Neubauer chamber and used for BUVEC infection.

### Cholesterol visualization and quantification

For staining intracellular stages for cholesterol, BUVEC were grown on coverslips (*n* = 3) and infected with *B. besnoiti* tachyzoites (MOI = 5:1). At 24 h p. i. (hours post infection), the samples were washed with PBS, fixed in 4% paraformaldehyde (10 min), washed three times with PBS. To detect free cholesterol, the samples were stained by filipin III (35 µg/mL in PBS, 15 min, in the dark, RT; Sigma-Aldrich). All samples were washed with PBS, mounted in Fluoromount-G mounting medium (Invitrogen). Cells were analysed using an inverted fluorescence microscope (IX81, Olympus) applying the UV filter set (340–380 nm excitation, 430 nm pass filter) and/or by using confocal microscopy analysis (Confocal LSM 710; Zeiss; 63X magnification, numerical aperture 1.2 µm). Single cells (*n* = 20) fluorescence intensity measurements were performed using ImageJ^37^ (mean grey value) and expressed as mean ± standard deviation. Image processing was carried out with ImageJ using merged channels plugins and restricted to minor adjustment of brightness and contrast.

Total cholesterol quantification was performed according to previous studies^27^. Therefore, total lipid extractions from *B. besnoiti*-infected (12, 24 and 48 h p. i.) and control BUVEC (*n* = 6) were executed in hexane:isopropanol^66^. The cells were washed twice with ice-cold PBS, trypsinized, washed again (400 × *g*, 10 min) and total cell numbers were counted using a Neubauer chamber. Hexane:isopropanol (3:2, *v*/*v*) was added to the cell pellet. Cells were disrupted for 10 min in Tissue Lyser (Qiagen) using stainless steel beads. After centrifugation (8,000 × *g*, 1 min) the supernatants were collected. The extraction was repeated once for each sample. Combined supernatants were dried manually under liquid nitrogen stream. The total lipid extracts were reconstituted in 500 µL isopropanol:NP40 (9:1; all Roth) and sonicated in a water bath (RT, 30 min). 5 µL of each sample were pre-treated with catalase [(5 µL of 0.5 mg/mL; Sigma-Aldrich) in 40 µL of 1X reaction buffer (37 °C, 15 min)] in 96-well black clear-bottom plates (Greiner Bio-One) to reduce background fluorescence of peroxides in the solvents^67^, before the enzyme cocktail of the Amplex Red Cholesterol Assay Kit (Life Technologies) was added. 50 µL of enzyme mixture (0.1 M potassium phosphate buffer, pH 7.4; 0.25 M NaCl, 5 mM cholic acid, 0.1% Triton X-100, 0.3 U/mL cholesterol oxidase, cholesterol esterase, 1.3 U/mL HRP, and 0.4 mM ADHP) were added and incubated at 37 °C for 15 min. Cholesterol standard (10, 5, 2.5, 1.25, 0.625 and 0.325 µM; Sigma-Aldrich) and blanks (solvent only) were included in every experiment. Resorufin formation was measured by fluorescence intensities (excitation wavelength of 530 nm, emission wavelength of 580 nm) in the Varioskan Flash Multimode Reader (Thermo Scientific). Total cholesterol of the samples was extrapolated to the values of the cholesterol standard and the total cholesterol content of each sample was normalized to its total cell number counts.

### Lipid droplet and neutral lipid staining and quantification

For staining of intracellular stages (24 h p. i.), BUVEC were grown on coverslips and infected, whilst tachyzoite stages were directly dropped onto poly-_L_-lysine-coated coverslips. Specimens were washed in PBS, fixed in 4% paraformaldehyde (10 min, RT), washed thrice in PBS and incubated in 1% glycine PBS (10 min, RT) to quench non-specific signals, followed by three washes in PBS. For neutral lipid and lipid droplet visualization, cells were stained with Nile Red (10 µg/ml, Sigma-Aldrich, 10 min, 37 °C, in the dark) or with Bodipy 493/503 (1 µg/mL, Life Technologies; 10 min, RT, in the dark), respectively. All samples were washed with PBS, mounted in Fluoromount-G mounting medium (Invitrogen) and analysed using an inverted fluorescence microscope (IX81, Olympus). Single cell (*n* = 20) fluorescence intensity measurements were performed using ImageJ^37^ (mean grey value) and expressed as mean ± standard deviation. Image processing was carried out with ImageJ using merged channels plugins and restricted to minor adjustment of brightness and contrast.

For neutral lipid quantification, infected BUVEC layers (MOI = 3:1) were stained with Nile Red. The mean fluorescence intensities per area of single infected (*n* = 20) and non-infected single cells (*n* = 20) were estimated applying identical experimental conditions using an inverted fluorescence microscope (IX81, Olympus). Fluorescence intensity measurements were performed using ImageJ^37^ (mean grey value) and expressed as mean ± standard deviation. For lipid droplet quantification, *B. besnoiti*-infected (MOI = 3:1 and 4:1) and control BUVEC (*n* = 3) were trypsinized at 12 and 24 h p. i. and pelleted (400 × *g*, 3 min, 4 °C). Resuspended cells were stained with Bodipy 493/503 (10 min, on ice) and washed twice with 1 ml PBS (400 × *g*, 3 min, 4 °C). The cells were transferred to 5-ml FACS tubes (BD Biosciences) containing 200 µl PBS and were processed in a FACSCalibur flow cytometer (BD Biosciences) by laser excitation at 488 nm (FL1-H channel). Flow cytometry data were acquired by the BD Cell Quest Pro software as previously reported (Hamid *et al*.^27^).

### Cholesterol and LDL supplementation experiments

For exogenous cholesterol supplementation, cholesterol and desmosterol (both Sigma-Aldrich) dissolved in ethanol^38^ were added to BUVEC cultures (*n* = 3) at 5 µM final concentration at the time point of *B. besnoitia* infection. Additionally, cholesterol enrichment was performed by supplementation of cholesterol-MβCD (Chol-MβCD; Sigma-Aldrich)-complexes in basal medium (PromoCell) lacking FCS (Chol-MβCD; 0.3 mM, 30 min, 24 h and 60 min prior infection). For the preparation of Chol-MβCD complexes (stock solution 10 mM), cholesterol was solved in MβCD water solution (40 mg/mL) at 30 °C, overnight, with constant agitation.

For LDL enrichment, non-modified LDL (Sigma-Aldrich, 10 mg/mL final concentration), acetylated LDL (acLDL; Life Technologies, 10 mg/mL final concentration) and oxidized LDL (oxLDL; Life Technologies, 2.5 mg/mL final concentration) were supplemented 24 h before *B. besnoiti* infection and ongoing until the end of the experiments.

To estimate the effect of supplementations on parasite proliferation, tachyzoite numbers were estimated 48 h p. i. by quantitative PCR.

### Lipid droplet enrichment

To artificially enhance lipid droplet formation in host cells, oleic acid (OA; Sigma-Aldrich) was supplemented in BSA formulation complexes to the cell culture medium according to Martin and Parton^68^. Direct conjugation was performed by mixing oleic acid-free BSA (fraction V, Roth) with oleic acid at the molar ratio of 6:1 (oleic acid:BSA). Prior to infection, an induction step was performed with culture medium suplemented with 50 µM OA (1 h, 37 °C, 5% CO_2_). Parasites were allowed to infect BUVEC in non suplemented cell culture medium for 4 h. Then, medium was changed and OA was suplemented in a final concentration of 2.5 µM.

### PCR-based quantification of *B. besnoiti* tachyzoites

Tachyzoite numbers were estimated via a quantitative PCR according to Cortes, *et al*.^69^. Therefore, biological triplicates with technical duplicates were processed. Firstly, cell culture supernatants containing free-released tachyzoites (=extracellular tachyzoites) were collected at 48 h p. i. and pelleted (600 × *g*, 15 min). In addition, the remaining host cells carrying not yet released tachyzoites (=intracellular tachyzoites) were trypsinized and pelleted (600 × *g*, 15 min). All cell/parasite pellets were treated with 200 μL of cell lysis buffer containing 0.32 M sucrose, 1% Triton X-100, 0.01 M Tris-HCl (pH 7.5), 5 mM MgCl_2_ and incubated in 100 μL 1X PCR buffer (Quanta) containing 20 μL proteinase K (20 mg/mL; Qiagen) at 56 °C for 1 h. Proteinase K was heat-inactivated by heating the samples (95 °C, 10 min) and the DNA-containing samples were frozen at −20 °C until further use. Real-time PCR was performed in a total volume of 20 μL containing 2 μL DNA of test samples, 400 nM of each primer, 200 nM probe and 10 μL PerfeCTa MasterMix (Quanta) at the following cycling conditions: 95 °C for 10 min; 40 cycles at 95 °C for 10 s, 60 °C for 15 s and 72 °C for 30 s.

### Biochemical estimation of cholesterol esterification and of cholesterol-related sterols

Pellets of *B. besnoiti*-infected BUVEC and of non-infected control cells were dried in a Savant SpeedVac concentrator (Thermo Fisher Scientific) for 24 h. Cholesterol, non-cholesterol sterols and oxysterols were extracted from dry weight aliquots using Folch reagent (chloroform/methanol; 2:1 (*v*/*v*); with 0.25 mg BHT added per mL solvent) per 10 mg dried cell pellets. Extraction was performed for 12 h at 4 °C in a dark cold room. The extracts were kept at −20 °C until further use. One mL of the Folch was submitted to alkaline hydrolysis, extraction of the free sterols and oxysterols, silylation to their corresponding (di)trimethylsilyl ethers prior to gas chromatographic separation and detection by mass selective detection (for non-cholesterol sterols or oxysterols using epicoprostanol and the corresponding deuterium labelled oxysterols as internal standards, respectively) as described in detail previously^70,71^. The degree of esterification of cholesterol was calculated from total (alkaline hydrolysis) and free cholesterol (without alkaline hydrolysis) concentrations using D6-cholesterol as internal standard.

### Inhibitor treatments

For inhibitor treatments, blockers of cholesterol esterification (CI976, Sigma-Aldrich), of fatty acid synthase (C75, Sigma-Aldrich) and of HMC-CoA reductase (lovastatin, Sigma-Aldrich) were used at concentrations of 2.5, 5, 10 and 20 µM^72^. Additionally, zaragozic acid (inhibitor of squalene synthase) was used at 15, 30 and 60 µM concentration. Viability assays were performed with non-infected BUVEC (*n* = 3) for all inhibitors in all mentioned concentrations for 72 h (CyQUANT XTT Cell Viability Assay, Invitrogen; Supplementary Fig. 3), according to manufacture instructions. For blocking experiments, BUVEC (*n* = 3) were grown to 80% confluency. Inhibitors were supplemented to the cell culture medium 24 h prior to parasite infection and from 4 h p. i. ongoing. For *B. besnoiti* infections, the medium was removed, BUVEC were washed once with PBS and tachyzoites were added to the cells (MOI = 4:1) in inhibitor-free medium. Four h p. i. the medium was removed and replaced by inhibitor-supplemented medium. 48 h after infection, the numbers of tachyzoites being present in cell culture supernatants were counted in a Neubauer chamber. Non-treated (medium only) and solvent (DMSO, acetone or ethanol)-treated, *B. besnoiti*-infected BUVEC were equally processed and served as negative controls.

### RT-qPCR for the relative quantification of LDLR andLOX-1 mRNAs

BUVEC (*n* = 3) grown in 25 cm^2^ culture tissue flasks were infected with freshly isolated *B. besnoiti* tachyzoites (MOI = 5:1). *B. besnoiti*-infected and control BUVEC were equally processed for total RNA isolation at different time points of parasite proliferation (3, 6, 12, 24 h p. i.). For total RNA isolation, the RNeasy kit (Qiagen) was used according to manufacturer’s instructions. Therefore, BUVEC were lysed within the cell culture flasks with RLT lysis buffer (600 µL/25 cm^2^ flask) and processed as proposed by the manufacturer including an on-column DNase treatment. Total RNAs were stored at −20 °C until further use. The quality of total RNA samples was controlled on 1% agarose gels. In order to remove any genomic DNA leftover, a second DNA digestion step was performed. Therefore, 1 µg of total RNA was treated with 10 U DNase I (Thermo Scientific) in 1x DNase reaction buffer (37 °C, 1 h). DNase was inactivated by heating the samples (65 °C, 10 min). The efficiency of genomic DNA digestion was verified by including no-RT-controls in each RT-qPCR experiment. cDNA synthesis was performed using the SuperScript III First-Strand Synthesis System (Thermo Fisher Scientific) according to manufacturer’s instructions with slight modifications. 1 μg of DNase-treated total RNA was added to 0.5 µL of 50 μM oligo d(T), 1 µL of 50 ng/μl random hexamer primer, 1 µL of 10 mM dNTP mix and DEPC-treated water was adjusted to 10 µL total volume. The samples were incubated at 65 °C for 5 min and then immediately cooled on ice. For first strand cDNA synthesis, 2 µL of 10x RT buffer, 4 µL 25 mM MgCl_2,_ 2 µL 0.1 M DTT, 1 µL RNaseOUT (40 U/µL, Thermo Fisher Scientific), 0.5 µL SuperScript III enzyme (200 U/µL) and 0.5 µL DEPC-treated water were added and the samples were incubated at 25 °C for 10 min followed by 50 °C for 60 min and a 85 °C-inactivation step for 15 min.

Primers (MWG Biotech) and probes used for qPCR are shown in Table 2^27^. Probes were labelled at the 5′-end with a reporter dye FAM (6-carboxyfluorescein) and at the 3′-end with the quencher dye TAMRA (6-carboxytetramethyl-rhodamine). qPCR amplification was performed on a Rotor-Gene Q Thermocycler (Qiagen) in duplicates in a 10 µL total volume containing 400 nM forward and reverse primers, 200 nM probe, 10 ng cDNA and 5 µL 2x PerfeCTa qPCR FastMix (Quanta Biosciences). The reaction conditions for all systems were as follows: 95 °C for 10 min, 40 cycles at 95 °C for 10 s, 60 °C for 15 s and 72 °C for 30 s. No-template controls (NTC) and no-RT reactions were included in each experiment. As reference gene GAPDH was used as previously reported^27,65,73^. Analyses of the qPCR data used the comparative ΔΔ*C*_T_ method^74^ and reported as n-fold differences comparing *B. besnoiti*-infected BUVEC with non-infected controls after normalizing the samples by the GAPDH reference gene.

**Table 2:** Sequences of primers and probes used in real-time qPCR.

Symbol	Name	Accession number	Amplicon length	Forward (5′ → 3′)	Reverse (5′ → 3′)	Probe (reporter 5′-3′quencher)
ACAT1	Acetyl-CoA acetyl-transferase 1	NM_001046075.1	102	TCATATGGGCAACTGTGCTGA	CTGCTTTACTTCTGGTATAG	FAM-AGCATAAGTATCCTGTTCCTCTCGTG-BHQ1
ACAT2	Acetyl-CoA acetyl-transferase 2	NM_001075549.1	194	AGCAGTGGTTCTTATGAA	AGGCTTCATTGATTTCAAA	FAM-ATCAACATCCTCCAGCGACCA-BHQ1
CH25H	Cholesterol 25-hydro-xylase	NM_001075243.1	87	TTGGGTGTCTTTGACATG	CAGCCAGATGTTGACAAC	FAM-CGTCTTGCTGCTCCAGTGTC-BHQ1
HMGCS	3-hydroxy-3-methylglutaryl-CoAsynthase 1	NM_001206578	197	CTACCTCAGTGCATTAGA	CTCTGTTCTGGTCATTAAG	HEX-AAGTCATTCAGCAACATCCGAGC-BHQ1
HMGCR	3-hydroxy-3-methylglutaryl-CoAreductase	NM_001105613.1	109	GCCATCAACTGGATAGAG	CCTCAATCATAGCCTCTG	FAM-TCTCTGACAACCTTGGCTGGAAT-BHQ1
LDLR	Low density lipoprotein receptor	NM_001166530.1	96	CGCCTACCTCTTCTTTAC	ACCACGTTCTTAAGGTTG	FAM-TCGCTTCGGTCCAGAGTCATC-BHQ1
OLR1	Oxidized low density lipoprotein/(lectin-like) receptor 1	NM_174132.2	102	CGCCTACCTCTTCTTTAC	ACCACGTTCTTAAGGTTG	TET-TCGCTTCGGTCCAGAGTCATC-BHQ1
SQLE	Squalene epoxidase	NM_001098061.1	132	CCCTTCTTCACCAGTAAA	CCCTTCAGCAATTTTCTC	HEX-AACAACAGTCATTCCTCCACCAGTA-BHQ1
GAPDH	Glyceraldehyde-3-phosphate dehydrogenase	AF-022183	82	GCGACACTCACTCTTCTACCTTCGA	TCGTACCAGGAAATGAGCTTGAC	FAM-CTGGCATTGCCCTCAACGACCACTT–BHQ1

### Protein extraction

For protein extraction, *B. besnoitia*-infected and non-infected BUVEC isolates (*n* = 3) were washed in PBS to remove any medium traces, trypsinized and pelleted (600 × *g*, 10 min). Proteins were extracted by homogenizing the cell pellets in RIPA buffer [50 mM Tris-HCl, pH 7.4; 1% NP-40; 0.5% Na-deoxycholate; 0.1% SDS; 150 mM NaCl; 2 mM EDTA; 50 mM NaF (all Roth)] in the presence of a protease inhibitor cocktail (Sigma-Aldrich). The homogenates were centrifuged at 10,000 × *g* for 10 min at 4 °C to sediment intact cells and nuclei. The supernatants were stored at −80 °C until further use. The protein content was quantified via Coomassie Plus (Bradford) Assay Kit (Thermo Scientific) following the manufacturer instructions.

### SDS-PAGE and Western Blotting

The samples were denatured using 6 M urea loading buffer and heated for 5 min at 95 °C. Fifty µg of total protein were loaded per slot and run in 12% polyacrylamide gels (120 V, 1.5 h)^75^. After electrophoretic separation, the proteins were transferred to a polyvinylidene difluoride (PVDF) membrane (Millipore) (200 mA, 2 h). Blots were blocked with 3% BSA in TBS containing 0.1% Tween (Sigma) for 1 h at RT and then incubated overnight in primary antibody solutions (see Table 3). Vinculin expression was used as a reference for the normalization of the samples. After washing in TBS containing 0.1% Tween (thrice, 5 min), blots were incubated in secondary antibody solutions (30 min, RT). Signal development was accomplished with an enhanced chemiluminescence detection system (ECL plus kit, GE Healthcare) and signal strength was determined in a ChemoCam Imager (Intas Science Imaging). Protein sizes were controlled by a protein ladder (PageRuler Plus Prestained Protein Ladder ~10–250 kDa, Thermo Fisher Scientific).

**Table 3:** List of antibodies used for Western blot and surface expression assays.

	Antibody	Company	Cat. number	Isotype	Dilution
Primary antibodies	Vinculin	Santa Cruz	sc-73614	Mouse	1:1,000
	ACAT-1	Sigma-Aldrich	AV54278	Rabbit	1:100
	ACAT-2	Abcam	ab66259	Rabbit	1:250
	CH25H	Abcam	ab133933	Rabbit	1:250
	LDLR	Santa Cruz	Sc-18823	Mouse	1:500
Secondary antibodies	Goat anti-mouse IgG Peroxidase conjugated	Pierce	31430	Mouse	1:40,000
	Goat anti-rabbit IgG Peroxidase conjugated	Pierce	31460	Rabbit	1:40,000

### Quantification of LDLR and LOX-1 expression

The surface expression of LDLR and LOX-1 was estimated in infected and non-infected BUVEC applying a flow cytometry-based technique according to Hamid *et al*.^27^. Therefore, BUVEC were infected with *B. besnoiti* tachyzoites (MOI 5:1). One day before infection, the cells were cultured in medium with lipoprotein-deficient serum (LPDS, 10%, Sigma-Aldrich). For measurements, medium was removed and cells were detached using accutase (Sigma-Aldrich) treatment (37 °C, 5 min) after a washing with PBS. Cells were pelleted (400 × *g*, 5 min, 4 °C) and incubated in anti-LDLR monoclonal (1:25, RT, 1 h; Antibody Online, ABIN235770) or anti-LOX-1 polyclonal (1:500, RT, 1 h Bioss Antibodies, bs-2044R) antibody solutions. After centrifugation (400 × *g*, 5 min, 4 °C), the cells were washed twice in PBS/0.01% NaN_3_ and incubated in secondary antibody solutions (1:40,000, 30 min, in the dark; Table 2). Secondary antibody controls were included in each experiment for signal normalization. After incubation, cells were washed thrice (400 × *g*, 5 min, 4 °C), resuspended in 100 μL PBS, transferred to 5 mL-FACS tubes (Greiner Bio-One) containing 200 μL of 1x PBS and processed in a FACSCalibur™ flow cytometer [Becton-Dickinson, Heidelberg, Germany; FL1-H channel (red)]. Data were acquired using the Cell Quest Pro (Becton-Dickinson) software.

Additionally, the expression of LOX-1 in *B. besnoiti*-infected BUVEC and non-infected control cells was determined by a commercially available bovine ELISA kit (DL-Develop). Therefore, BUVEC (*n* = 3) were grown to subconfluency in 75 cm^2^ cell culture flasks (Greiner) and infected at an MOI of 5:1 with freshly collected *B. besnoiti* tachyzoites. Cells were harvested at different time points p. i. (12, 24, 36 h p. i.) according to manufacturer’s instructions. Briefly, after washing with PBS, the cells were trypsinized and pelleted (400 × *g*, 12 min). Cell pellets were washed thrice with PBS and the number of cells per sample was determined microscopically before ultrasonication treatment (3 times for 20 s, on ice). Samples were centrifuged at 1,000 × *g* (15 min, 2–8 °C) to remove cell debris and the supernatants were stored at −20 °C until being processed by the ELISA kit.

### Live cell 3D holotomographic microscopy

BUVEC (*n* = 3) were seeded into 35 mm tissue culture µ-dishes (Ibidi^®^), grown overnight and infected with freshly released *B. besnoiti* tachyzoites (MOI 3:1). At 24 h p. i., holotomographic images were obtained by using 3D cell-explorer microscope (Nanolive 3D) equipped with a 60x magnification (λ = 520 nm, sample exposure 0.2 mW/mm^2^) and a depth of field of 30 µm^76^. For lipid droplet visualization, cells were stained with Bodipy 493/503 (1 µg/mL, Life Technologies; 3 h, 37 °C, in the dark). After incubation, medium was changed. Live cell 3D holotomographic microscopy and analysis of Bodipy 493/503-based fluorescence was performed in parallel to prove the identity of lipid droplets. A total of 50 lipid droplets were measured for their refractive index to obtain marker values for these organelles in BUVEC. Images were analysed using STEVE software (Nanolive) to obtain a refractive index-based z-stack^77^ and digital staining was applied according to the refractive index of the lipid droplets.

### Statistical analysis

Statistical analyses were performed with the statistical program package BMDP^78^ or with GraphPad Prism. In all cases, data revealed as normally or log-normally distributed (verified by residual analysis) and parametric statistical methods could be applied. Data description was performed by presenting arithmetic mean ± standard deviation for not log-transformed data, by geometric mean and standard deviation for log-transformed data or as n-fold changes relative to the controls. Depending on the design of the experiment, some data were analysed by one- or two-way analysis of variance (ANOVA) with repeated measures (program BMDP2V) to test the effects of infection and/or incubation time, dose of the inhibitor or cell ratio. If only one time point was considered, the analysis could be reduced to a t-test for dependent samples (program BMDP3D). In the case of a hierarchical design of the experiments incorporating more than one random factor (e. g. BUVEC and replication) a general mixed model analysis (glmm) with equal sample size (program BMDP8V) was applied. Occasionally, post hoc pairwise multiple comparison tests succeeded the global comparison by ANOVA, being performed either by Student-Newman-Keuls method (SNK-test) or by Bonferroni-Holm method controlling the family-wise error rate, or even by*t*-test. The outcomes of the statistical tests were considered to indicate significant differences when *p* ≤ 0.05 (significance level).

## Supplementary information

Supplementary Information

## Data Availability

All data generated or analysed during this study are included in this published article (and its Supplementary Information Files).
