# Supplementary material for: Besnoitia besnoiti infection alters both endogenous cholesterol de novo synthesis and exogenous LDL uptake in host endothelial cells
Source: Sci Rep. 2019 Apr 30;9:6650. doi: 10.1038/s41598-019-43153-2 (PMC6491585; doi:10.1038/s41598-019-43153-2)

Supplementary Information

Besnoitia besnoiti infection alters both endogenous cholesterol *de novo* synthesis and exogenous LDL uptake in host endothelial cells

Liliana M. R. Silva, Dieter Lütjohann, Penny Hamid , Zahady D. Velasquez, Katharina Kerner, Camilo Larrazabal, Klaus Failing, Carlos Hermosilla, Anja Taubert

Figure S1. Immunofluorescence analysis of filipin III. BUVEC monolayers (*n* = 3) were stained with filipin and single cell fluorescence intensity measurements were performed comparing non-infected (orange arrows) with *B. besnoiti*-infected (white arrows) cells. (**A**) Phase contrast, (**B**) filipin III and (**C**) merge.

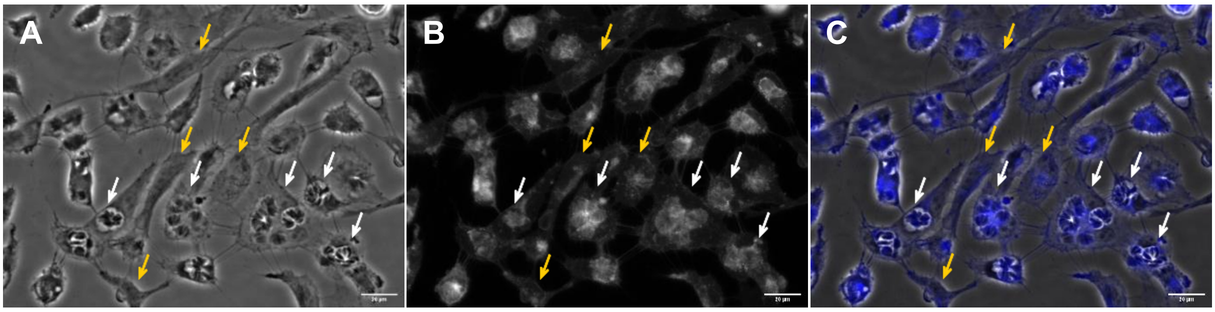

Figure S2. Immunofluorescence analysis of Nile Red. BUVEC monolayers ($n = 3$) were stained with Nile Red and single cell fluorescence intensity measurements were performed comparing non-infected (orange arrows) with *B. besnoiti*-infected (white arrows) cells. (**A**) Phase contrast, (**B**) Nile Red and (**C**) merge.

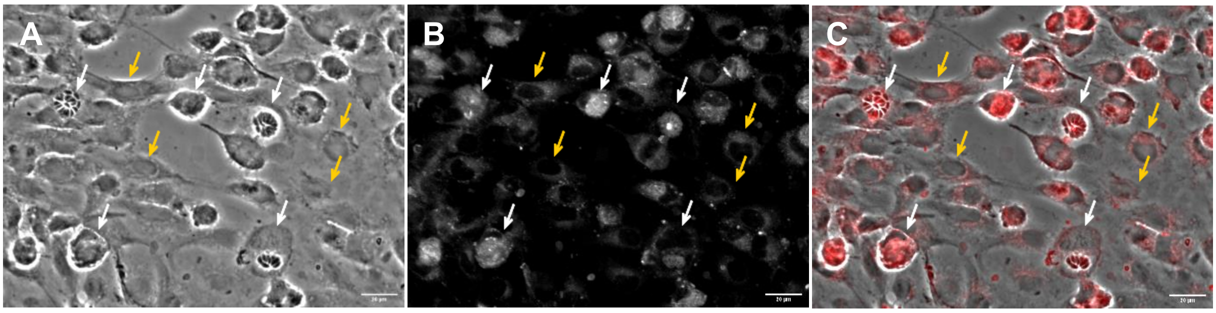

Figure S3. Viability assays performed with all used inhibitors in non-infected BUVEC and their effect on cell viability after 72h incubation. (**A**) Solvent controls, (**B**) CI976, (**C**) C75, (**D**) lovastatin and (**E**) zaragozic acid.

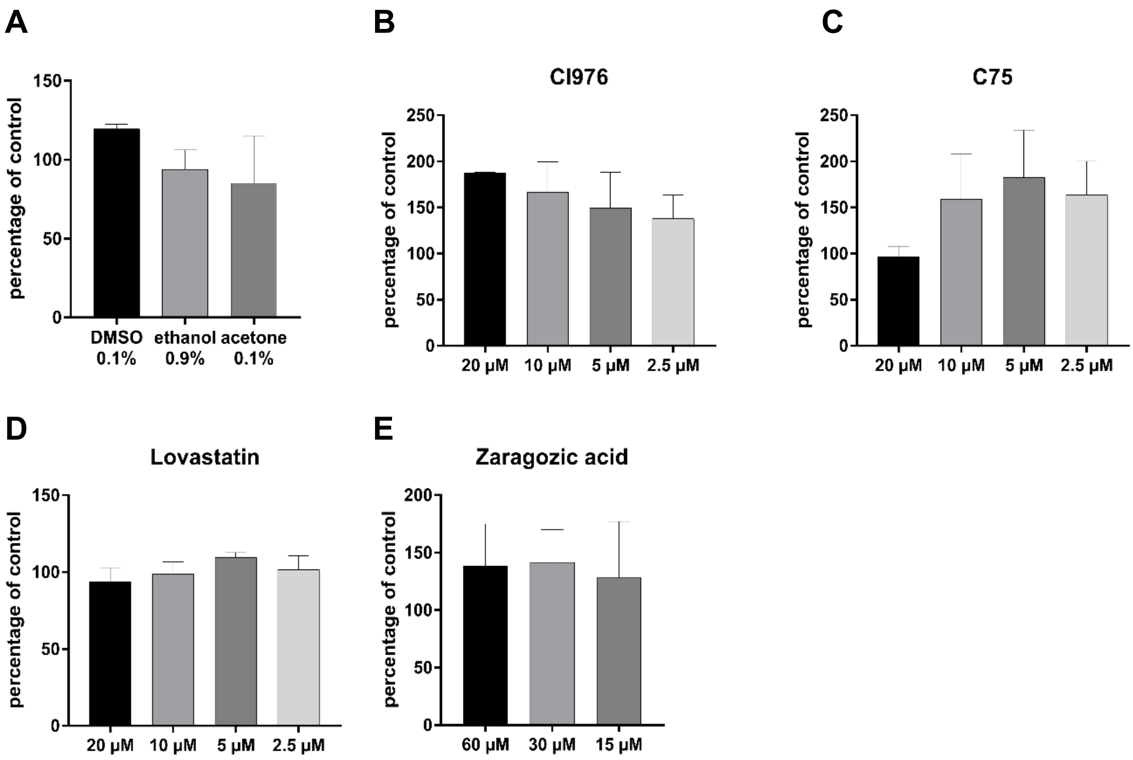

Figure S4. Raw data of immunoblots. (**A**) Original un-cropped Western blot detecting LDLR (92 kDa). (**B**) Original Western blot detecting vinculin (130 kDa). This membrane was cropped before immunodetection and assayed for proteins of different masses. The blots in (**A**) and (**B**) were cropped and shown in the Figure 6C.

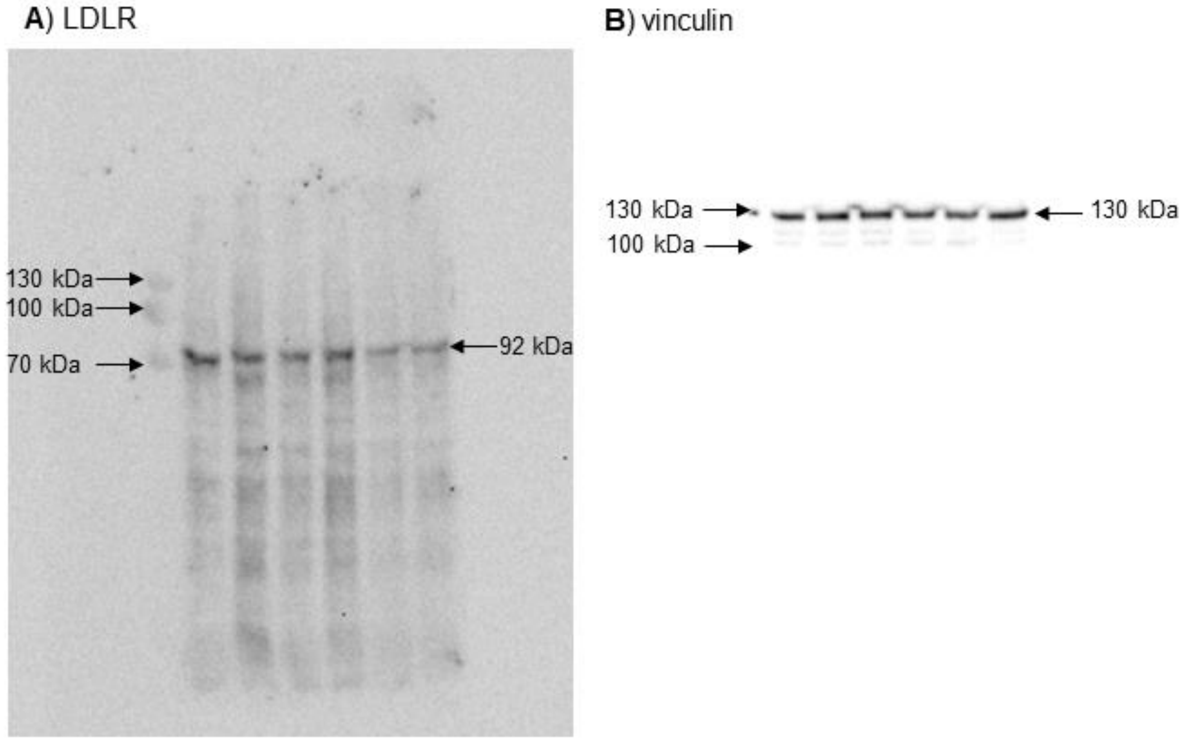

Supplement: Supplementary file 1 — Supplementary Information [file 41598_2019_43153_MOESM1_ESM.pdf]
